# Liquid–Liquid Phase Separation in Viral Infection and Immunology

**DOI:** 10.1002/mco2.70674

**Published:** 2026-03-24

**Authors:** Jiuzhi Xu, Lan Bai, Bin Wang, Hai Song, Long Zhang, Fangfang Zhou

**Affiliations:** ^1^ Department of Respiratory and Critical Care Medicine Center For Oncology Medicine The Fourth Affiliated Hospital of School of Medicine and International School of Medicine International Institutes of Medicine Zhejiang University Yiwu China; ^2^ Zhejiang Key Laboratory of Precision Diagnosis and Treatment for Lung Cancer Zhejiang‐Sweden Joint Laboratory On Tumor Immunology Yiwu China; ^3^ MOE Laboratory of Biosystems Homeostasis & Protection and Innovation Center For Cell Signaling Network Life Sciences Institute Zhejiang University Hangzhou China; ^4^ Department of Infectious Diseases Children's Hospital Affiliated to Soochow University Soochow University Suzhou China; ^5^ Department of Radiation Oncology and the State Key Laboratory of Transvascular Implantation Devices The Second Affiliated Hospital of Zhejiang University School of Medicine, Life Sciences Institute Zhejiang University Hangzhou China; ^6^ The MOE Basic Research and Innovation Center for the Targeted Therapeutics of Solid Tumors, The First Affiliated Hospital Jiangxi Medical College Nanchang University Nanchang China; ^7^ School of Medicine Zhejiang University City College Hangzhou China; ^8^ Institutes of Biology and Medical Science Soochow University Suzhou China

**Keywords:** antiviral therapy, biomolecular condensates, immunity, LLPS, viral infection

## Abstract

Liquid–liquid phase separation (LLPS) has emerged as a fundamental physicochemical principle that organizes macromolecules into dynamic, membraneless condensates. These assemblies are increasingly recognized as critical regulators of diverse cellular processes. Notably, both viruses and their hosts exploit LLPS to optimize their respective strategies for replication and defense, forming a dynamic interplay centered around phase separation. However, a comprehensive mechanistic understanding of how LLPS modulates the dynamic viral–host battle, and how this knowledge can be leveraged for therapeutic development, remains an active area of investigation. This review systematically explores the dual roles of LLPS in viral infection and antiviral immunity. We detail how viruses hijack LLPS to form replication factories and inclusion bodies that enhance entry, replication, and immune evasion. Conversely, we explore how host cells leverage LLPS to assemble potent immune signaling hubs, such as those nucleated by cGAS–STING, NLRP6 inflammasomes, and T/B‐cell receptor microdomains, to amplify antiviral responses. Furthermore, we critically evaluate emerging therapeutic strategies that target these phase separation interfaces. By integrating recent advances across virology, immunology, and biophysics, this review establishes a unified framework for understanding and targeting LLPS in viral infectious diseases, offering new perspectives for future basic research and clinical intervention.

## Graphical abstract

LLPS organizes viral replication and antiviral immunity. Viruses hijack LLPS to form replication factories and evade immune sensors, while hosts assemble LLPS‐driven signaling hubs (e.g., MAVS, RIG‐I, and SGs) to amplify interferon responses. Targeting these condensate interfaces offers novel therapeutic strategies against infectious diseases.

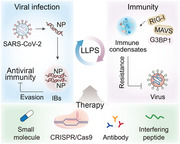

## 1 Introduction

**FIGURE 1:**
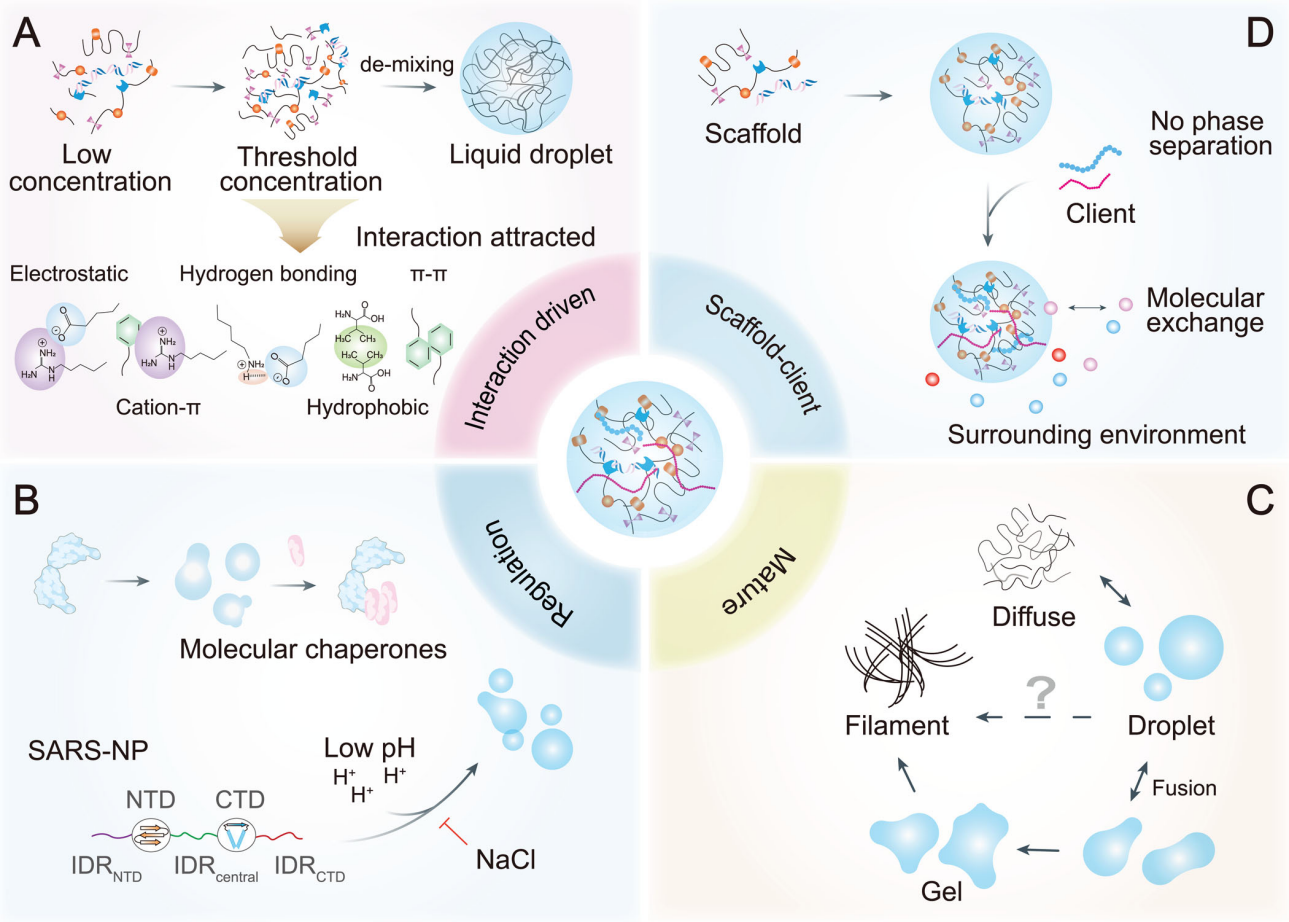
Basic principles of liquid–liquid phase separation (LLPS). (A) While entropy typically compels molecules to disperse in solution at low molecular concentrations, increasing the concentration enhances the multivalent interactions (e.g., electrostatic, cation–π, H‐bonding, hydrophobic, π–π) among molecules to exceed a critical threshold, thereby shifting the free energy landscape. This shift favors demixing and facilitates LLPS, resulting in the formation of a separate condensed phase with liquid‐like properties. (B) LLPS is regulated by both environmental and cellular factors. For example, molecular chaperones aid condensate assembly, and electrostatic forces affect the RNA‐induced LLPS of SARS‐NP. (C) LLPS‐derived condensates exhibit varied material states, ranging from liquid droplets to gels and filaments. (D) Phase‐separating biomolecules are typically classified as scaffolds and clients. Scaffolds are both necessary and sufficient to drive phase separation on their own. In contrast, clients selectively partition into condensates formed by scaffolds but cannot undergo phase separation independently. The liquid‐like properties of these condensates allow for dynamic molecular exchange with the surrounding environment.

Eukaryotic cells orchestrate their complex activities in a spatiotemporally controled manner through a dual architectural system, comprising both membrane‐bound organelles and membraneless compartments. The latter, known as biomolecular condensates, include stress granules (SGs), processing bodies (P bodies), and various nuclear speckles, which assemble via a process called liquid–liquid phase separation (LLPS) [1, 2, 3]. LLPS is a physicochemical phenomenon in which biomolecules (such as proteins and nucleic acids) spontaneously separate from a homogeneous solution into dense, liquid‐like condensates and a surrounding dilute phase. This demixing is driven by weak, multivalent interactions between modular domains and intrinsically disordered regions (IDRs) (Figure 1A). Seminal studies by Brangwynne et al. on P granules in *C. elegans* embryos first demonstrated the biological relevance of LLPS, establishing it as a fundamental principle of subcellular organization beyond classical membrane‐bound compartments. Subsequent research has established that LLPS governs the formation of diverse cellular structures such as nucleoli, nuclear pores, and signaling hubs, implicating it in transcription, RNA processing, and cellular stress response. Dysregulation of LLPS is implicated in various pathologies, including neurodegenerative diseases and cancer [4, 5].

The dynamic and reversible nature of LLPS allows cells to rapidly assemble and disassemble functional condensates in response to stimuli. This property is strategically exploited by viruses to facilitate their replication and evade host immunity. The composition and material states of LLPS‐driven condensates, which can range from liquid to gel‐like or solid, are governed by molecular concentration, posttranslational modifications (PTMs), and environmental cues. These properties enable LLPS to support both physiological adaptability and pathological aggregation, as observed in neurodegenerative diseases and cancer (Figure 1B,C) [6, 7, 8].

Recently, LLPS has emerged as a key regulatory mechanism at the virus–host interface. Viruses, as obligate intracellular parasites, exploit host LLPS to form viral replication factories (RFs) or inclusion bodies (IBs), which are specialized condensates that concentrate viral components while excluding or inactivating host antiviral sensors. For instance, the nucleocapsid protein of SARS‐CoV‐2 (SARS2‐NP) undergoes LLPS to assemble replication‐competent condensates that shield viral RNA from detection by sensors such as RIG‐I and MDA5. Conversely, the host immune system utilizes LLPS to amplify antiviral signaling. Key immune sensors, including MAVS, STING, and cGAS, form LLPS‐driven signaling hubs that potentiate interferon (IFN) and inflammatory responses with enhanced specificity and temporal control. In adaptive immunity, phase separation also facilitates the clustering of T‐cell and B‐cell receptors (BCRs) into microdomains that drastically enhance signal transduction upon antigen recognition, thereby laying the foundation for robust specific immunity [9, 10, 11, 12, 13, 14, 15].

Despite these significant advances, critical questions remain unresolved: First, how do viruses selectively recruit host factors into viral condensates while evading immune sensors? Second, what determines the functional specificity of viral versus immune condensates? Third, can LLPS be therapeutically targeted to disrupt viral replication without harming host physiological condensates? Current literature lacks a comprehensive review that integrates mechanistic insights across diverse viral families with a systematic discussion of these therapeutic frontiers [16, 17].

This review aims to establish a unified framework for understanding LLPS in the context of viral infectious diseases. We begin by elucidating the biophysical principles of LLPS, then analyze how diverse viruses hijack LLPS across their lifecycle, from entry to egress. Subsequently, we discuss how host innate and adaptive immune systems leverage LLPS to mount defenses. Notably, we critically evaluate emerging therapeutic strategies that target the LLPS interface. Finally, we outline future perspectives and challenges in this rapidly evolving field.

## 2 Basic Principles of LLPS

LLPS is a fundamental physicochemical process in which biomolecules, primarily multivalent proteins and nucleic acids, spontaneously separate from a homogeneous aqueous solution to form dense, liquid‐like condensates that coexist with a surrounding dilute phase. This demixing is governed by a balance between entropic forces favoring dispersal and enthalpic contributions from multivalent interactions, which collectively drive the system beyond a critical concentration threshold into a two‐phase regime [1, 18]. These condensates, often referred to as biomolecular condensates, act as dynamic, membraneless organelles (MLOs) that organize diverse cellular processes, including transcription, RNA processing, and signal transduction.

### 2.1 Molecular and Energy Basis for LLPS

The formation and stability of biomolecular condensates depend on a network of weak, transient interactions, including electrostatic forces, cation–π, π–π, cation–anion, and hydrophobic effects [1, 5, 19, 20]. These interactions are often mediated by modular protein domains and IDRs, which provide the multivalency needed to overcome the entropic penalty of phase separation. Within condensates, biomolecules can be functionally classified as scaffolds or clients [21]. Scaffold proteins, typically enriched in IDRs or repetitive modular domains (RMDs), possess an innate ability to drive LLPS through their multivalent interaction platforms [6, 20]. In contrast, client molecules lack independent phase‐separating capacity but are selectively recruited into condensates through specific affinities for scaffold components, enabling context‐dependent regulation of condensate composition and function (Figure 1D) [6, 22].

#### 2.1.1 Intrinsically Disordered Regions

IDRs are flexible polypeptide segments that lack stable folded structures but are enriched in low‐complexity sequences (e.g., arginine/glycine/tyrosine‐rich motifs). Their conformational plasticity allows rapid, reversible assembly into condensates, making them ideal for dynamic cellular organization [22, 23, 24, 25, 26, 27, 28]. PTMs such as phosphorylation and methylation can finely tune the phase behavior of IDR‐containing proteins by altering their charge, hydrophobicity, or interaction valency. For example, the methylation state of FUS regulates its LLPS propensity, with consequences for both physiological function and pathological aggregation [29]. In antiviral immunity, sensors like RIG‐I and MDA5 exploit C‐terminal IDRs to form condensates that promote MAVS aggregation and enhance IFN responses upon viral RNA detection [30]. Similarly, the NLRP3 inflammasome assembles via phosphorylation‐modulated LLPS of its IDR, boosting caspase‐1 activation during infection [30, 31]. Building on these natural principles, Jin et al. have designed intrinsically disordered protein‐inspired, nanovector‐based coacervates for the direct cytosolic transport of biomacromolecules, illustrating how IDR‐driven phase separation can be harnessed for biomedical applications [32].

#### 2.1.2 Repetitive Modular Domains

RMDs, such as SH2, SH3, RRM, and WD40 repeats, provide multiple binding sites that increase valency and promote LLPS when engaged with cognate ligands. A classic example is polyubiquitin chains, which serve as multivalent scaffolds driving p62 phase separation during selective autophagy [21, 33]. During viral infection, TLR3 employs its TIR domain to recruit TRIF, forming signaling condensates that concentrate TBK1 and IRF3, thereby potentiating IFN production [34]. Conversely, herpesviruses exploit host RMD‐containing autophagy proteins (e.g., ATG16L1) to evade autophagic clearance [35, 36].

#### 2.1.3 Dimerization/Oligomerization Regions

Oligomerization domains substantially increase valency by promoting protein multimerization, thereby lowering the critical concentration required for LLPS and stabilizing condensates [22, 37]. For example, the C‐terminal dimerization domain of SARS‐NP is essential for RNA‐induced LLPS and viral ribonucleoprotein (vRNP) assembly [3]. Similarly, oligomerization of the HIV‐1 capsid protein drives viral core formation, underscoring the critical role of multimerization in viral pathogenicity [38, 39].

#### 2.1.4 Nucleic Acid Binding Regions

Nucleic acids further facilitate LLPS through electrostatic and stacking interactions with RNA‐binding domains, such as arginine‐rich motifs and RNA recognition motifs (RRMs) [40, 41, 42, 43]. Many RNA viruses, including HCV and IAV, exploit this mechanism to form replication compartments that concentrate viral components while excluding cytosolic immune sensors [44, 45, 46, 47]. Similarly, host defense systems leverage nucleic acid‐triggered LLPS; for example, cGAS undergoes DNA‐induced phase separation that enhances cGAMP synthesis and amplifies STING‐dependent IFN responses [48].

### 2.2 Dynamic Regulation and Functional Implications

LLPS is not a static endpoint but a highly regulated process in which condensates can mature from liquid‐like droplets to gel‐like or solid states. This dynamic continuum allows condensates to function as responsive signaling hubs or, when dysregulated, as seeds of pathological aggregation, as observed in neurodegenerative diseases and certain cancers. Regulation occurs through PTMs (e.g., phosphorylation, SUMOylation, acetylation), as well as changes in ionic strength, pH, light, temperature, and the activity of molecular chaperones, which collectively fine‐tune condensate formation, disassembly, and composition (Figure 1B) [32, 49, 50].

Such precise regulation enables biomolecular condensates to function as adaptable platforms that concentrate specific reactants, enhance biochemical reaction rates, and sequester inhibitory factors, functions that are co‐opted by both viral pathogens and host immune systems. The dysregulation of LLPS, whether through viral hijacking, genetic mutation, or age‐related decline in cellular quality control, can result in immune evasion, defective host responses, or disease. Given this dynamic nature, LLPS is also being developed for biomaterials like bioadhesives, microreactors, drug delivery systems, and optical signals, highlighting its broad potential for scientific research and clinical applications [51].

## 3 Role of LLPS in Viral Infection

**FIGURE 2:**
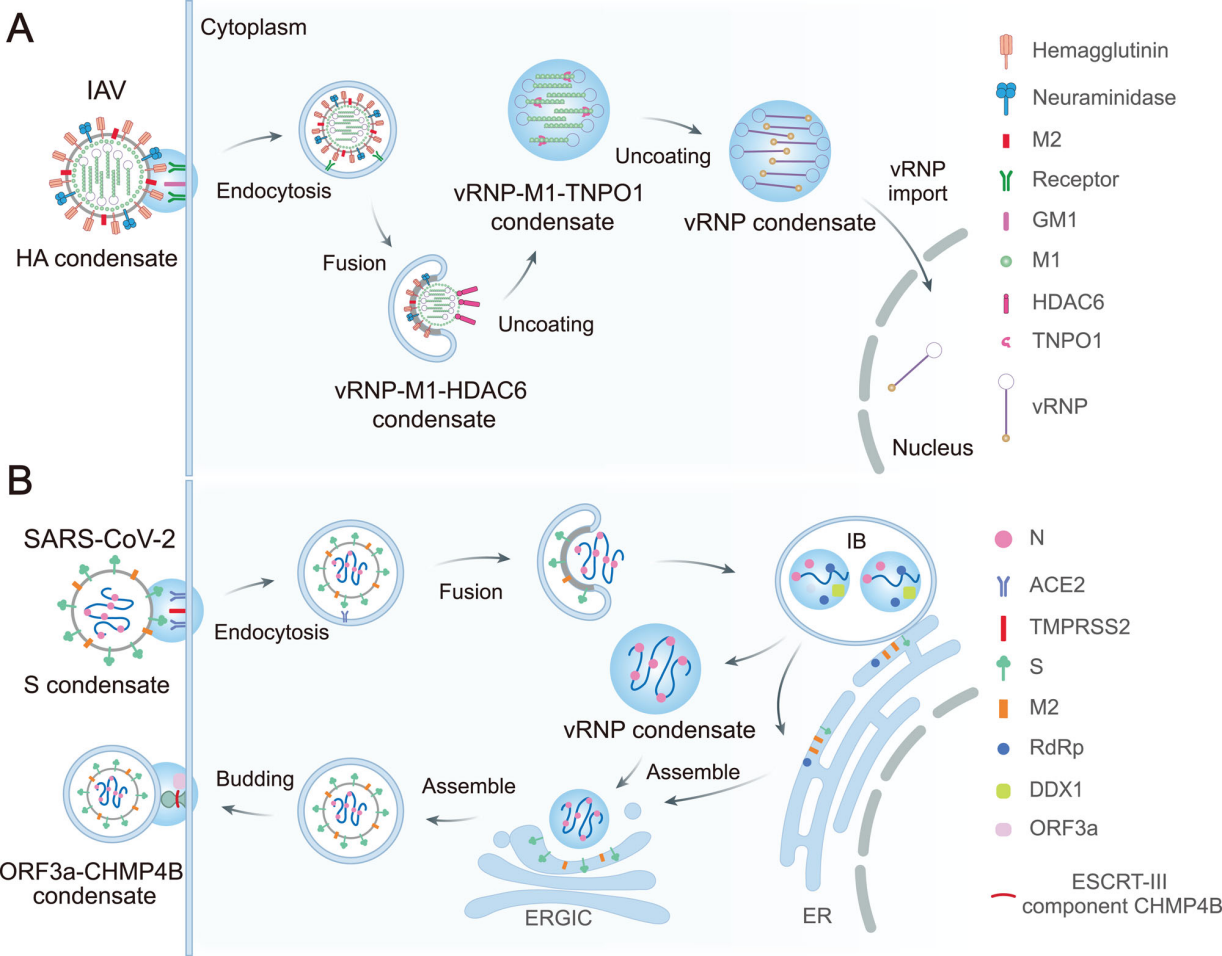
Role of LLPS in viral replication cycles. (A) Life cycle of the influenza A virus (IAV) begins when the hemagglutinin (HA) glycoprotein binds to sialic acid receptors on host membranes. HA‐enriched condensates boost binding avidity by increasing the local receptor density and promoting receptor‐mediated endocytosis. Subsequently, the fusion of viral and vesicle membranes releases viral RNA–protein (vRNP) complexes into the cytoplasm. In the condensate, vRNPs are uncoated through the action of HDAC6 and TONP1, allowing for the import of uncoated vRNPs into the nucleus. (B) The binding of SARS‐CoV‐2 particles to the ACE2 receptor at the plasma membrane is enhanced by spike‐enriched condensates. This enhanced binding leads to membrane fusion, which releases the positive‐stranded RNA genome into the cytoplasm. NPs and nucleic acids drive LLPS to form a specialized intracellular compartment called the inclusion body (IB), which functions as a replication factory. Newly produced N proteins phase‐separate from genomic RNA to form vRNP complexes. Thereafter, membrane‐bound structural proteins M, S, and E translocate to the plasma membrane via the ER–Golgi intermediate compartment (ERGIC). Viral particles are assembled, transported to the plasma membrane and released via exocytosis, which is dependent on the LLPS of ORF3a–CHMP4B.

Viruses have evolved to exploit LLPS as a versatile biophysical strategy for compartmentalizing their replication machinery, evading host immunity, and optimizing viral fitness [52]. This exploitation spans the entire viral lifecycle, from entry to egress, with LLPS‐driven condensates forming specialized microenvironments that concentrate viral components while excluding host defense factors. Understanding how different viral families leverage LLPS provides critical insights into viral pathogenesis and identifies potential vulnerabilities for therapeutic intervention (Figure 2A,B) [53].

### 3.1 Virus Entry and Uncoating

Viral entry is the first critical step in infection, where LLPS‐mediated spatial organization enhances both efficiency and immune evasion, a strategy conserved across diverse viral families. The initial stages of infection, including receptor engagement, membrane fusion, and genome release, are precisely regulated by this phase separation. Enveloped viruses particularly exploit LLPS to enhance entry efficiency while minimizing immune detection [54]. For IAV, the hemagglutinin (HA) glycoprotein undergoes LLPS via its transmembrane domain, which contains low complexity regions (LCRs) enriched in aromatic residues. This phase separation enables HA clustering into liquid‐ordered membrane domains, boosting local receptor density through multivalent interactions. Cryo‐electron tomography has shown that these HA‐enriched condensates form hexagonal lattices within phase‐separated lipid rafts, effectively reducing the energy barrier for membrane curvature induction and fusion pore formation [55]. Concurrently, host gangliosides undergo complementary phase separation, creating specialized lipid domains that colocalize with HA clusters and facilitate cholesterol‐dependent membrane remodeling [53, 56, 57].

The uncoating process represents another critical phase where LLPS plays a regulatory role. For IAV, acidic pH (∼5.0) in endosomes triggers conformational changes that dissolve LLPS‐stabilized M1–vRNP interactions. This dissolution is actively regulated by host histone deacetylase 6 (HDAC6), which binds ubiquitinated M1 and remodels vRNP IDRs through its deacetylase activity. Super‐resolution live imaging has demonstrated that HDAC6 recruitment induces rapid dissolution of M1 condensates, facilitating vRNP nuclear import [57, 58, 59, 60]. This import process is further refined by transportin‐1, which acts as a phase separation chaperone by competitively binding proline‐tyrosine nuclear localization signals on M1, thereby reducing interaction valency and ensuring precise spatiotemporal control of vRNP release (Figure 2A) [61, 62, 63, 64, 65, 66, 67].

The universality of LLPS‐driven entry strategies is evident across diverse viral families. HIV‐1 utilizes LCRs within the gp41 cytoplasmic tail to undergo LLPS with host tetraspanins, generating specialized entry portals enriched in the coreceptors CXCR4/CCR5 at lipid raft microdomains [68, 69]. Similarly, SARS‐CoV‐2 exploits LLPS at its furin cleavage site, where the RRAR motif phase separates with host proteases like TMPRSS2, locally concentrating enzymatic activity required to prime the spike protein for membrane fusion (Figure 2B) [70]. This evolutionary convergence on LLPS‐mediated entry mechanisms across unrelated viral families underscores its role as a fundamental organizing principle of viral entry. Consequently, targeting conserved phase‐separation events involved in receptor clustering, protease recruitment, and membrane remodeling may provide a conceptual framework for developing broad‐spectrum antiviral strategies [71, 72, 73].

### 3.2 Viral Replication and Transcription

During replication, viruses induce the formation of specialized intracellular compartments that balance efficient genome amplification with immune evasion. These LLPS‐driven structures, variously termed viral factories, IBs, or viroplasms, represent hallmark features of infection and display classic condensate properties, including dynamic exchange, fusion, and reversibility [74, 75]. The biophysical properties of these replication compartments vary widely across viral families, reflecting distinct replication strategies and modes of host manipulation.

Positive‐strand RNA viruses typically establish membrane‐associated replication complexes connected to organelles such as the endoplasmic reticulum or mitochondria, whereas negative‐sense RNA viruses often generate membrane‐less cytoplasmic inclusions that nonetheless maintain liquid‐like behavior [76, 77, 78, 79, 80, 81]. Ebola virus exemplifies a hierarchical LLPS mechanism driven by its nucleoprotein (NP). The N‐terminal RNA‐binding domain engages viral RNA through multivalent electrostatic interactions, while the C‐terminal dimerization domain promotes NP–NP oligomerization, forming liquid‐like IBs that function as molecular sieves. Early IBs exclude antiviral sensors such as RIG‐I and protein kinase R (PKR) through size‐dependent partitioning (pore size < 10 nm), creating immune‐privileged spaces for viral replication [82, 83, 84, 85, 86, 87]. As infection progresses, recruitment of the viral polymerase and cofactors (VP35, VP30) generates more complex architectures. VP35 crosslinks NP to form gel‐like scaffolds, and VP30 phosphorylation increases RNA‐binding valency, ensuring precise genome replication. Concurrently, hijacking of host helicase DDX3X creates dynamic subcompartments within IBs that enhance polymerase processivity by unwinding structured viral RNA [13, 84, 88, 89].

In SARS‐CoV‐2, PTMs‐regulated NP phase separation provides sophisticated spatiotemporal control over replication and packing. The NP's modular design, which comprises RNA‐binding LCRs, oligomerization domains, and IDRs, supports robust LLPS behavior. Hypophosphorylated NP forms liquid condensates with viral RNA that recruit the viral RdRp complex and host helicase DDX1, enhancing replication fidelity through molecular crowding effects [90, 91, 92, 93, 94]. A critical regulatory switch occurs through CK2‐mediated phosphorylation of serine/arginine‐rich motifs, which induces gelation and excludes RdRp while enriching packaging signals like RNA stem‐loop SL4. This controlled phase transition coordinates the shift from replication to assembly, ensuring selective genome packaging and efficient virion production [90, 95, 96].

### 3.3 Viral Assembly and Egress

LLPS coordinates the final stages of viral propagation through hierarchical assembly lines that package genomes and sculpt viral envelopes with remarkable precision. The assembly of SARS‐CoV‐2 exemplifies this sophisticated organization, progressing through nucleocapsid condensation, envelope scaffolding, and selective genome packaging. In the first stage, hypophosphorylated NP undergoes phase separation with genomic RNA to form vRNP complexes through multivalent interactions between its RNA‐binding domains and viral packaging signals. Fluorescence correlation spectroscopy reveals that these NP–RNA condensates selectively enrich genomic RNA while excluding nonspecific cellular RNAs, ensuring high‐fidelity packaging [91, 97, 98, 99, 100]. These vRNPs function both as structural precursors and protective containers that shield viral genomes from nucleases and innate immune sensors.

Subsequently, the membrane protein orchestrates envelope assembly at the ERGIC through its C‐terminal amphipathic helix, which promotes phase separation with anionic lipids such as PI4P/PIP2. The resulting liquid–crystalline matrix serves as a scaffold that recruits other structural proteins through charge complementarity and specific protein–protein interactions. This LLPS‐mediated organization is crucial for templating the characteristic spherical morphology of SARS‐CoV‐2 virions [95, 98, 101, 102, 103, 104, 105, 106]. The final packaging step is regulated by ATP‐dependent molecular chaperones, particularly HSP90, which localizes to the ERGIC and facilitates partial dissolution of M‐protein condensates. This creates transient openings that allow nucleocapsid envelopment, revealing the virus's ability to hijack cellular quality‐control machinery [107].

During viral egress, the ESCRT machinery facilitates membrane scission through LLPS‐mediated mechanisms. SARS‐CoV‐2 ORF3a induces phase separation of the ESCRT‐III component CHMP4B, forming constrictive rings at budding necks that mediate membrane scission and virion release (Figure 2B) [108, 109, 110]. HIV‐1 shows a similar strategy: its Gag protein phase separates with the ESCRT‐associated proteins ALIX and TSG101 to recruit ESCRT‐I/III complexes, and mutations in late Gag domains disrupt this interaction and impair viral release [111, 112]. This evolutionary convergence underscores the central importance of LLPS in viral egress across diverse viral families, suggesting that targeting these conserved phase separation processes could enable broad‐spectrum antiviral strategies.

## 4 LLPS in Antiviral Immunity

The host immune system has evolved to leverage LLPS as a powerful spatiotemporal regulatory mechanism to orchestrate rapid and potent antiviral responses. By concentrating signaling molecules into dynamic condensates, LLPS facilitates signal amplification, enhances specificity, and ensures timely immune activation. This section systematically examines how LLPS governs the assembly and regulation of key innate and adaptive immune signaling hubs, emphasizing the underlying biophysical principles and their functional consequences [113].

### 4.1 RNA Sensing Pathways

#### 4.1.1 RLR Pathway and SGs

The RIG‐I‐like receptor (RLR) pathway illustrates how LLPS converts cytosolic RNA detection into robust antiviral signaling. Upon recognizing viral RNA, RIG‐I and MDA5 undergo conformational changes that expose their caspase activation and recruitment domains, enabling oligomerization and interaction with E3 ubiquitin ligases such as TRIM25 for K63‐linked polyubiquitination [9, 114, 115]. This essential PTM facilitates the recruitment of MAVS, which forms prion‐like aggregates on mitochondrial membranes, a process now recognized as a terminal stage of LLPS. These MAVS fibrils constitute highly stable, proteolysis‐resistant condensates that irreversibly commit to downstream signaling, resulting in TBK1 activation, IRF3/IRF7 phosphorylation, and induction of Type I IFNs [116, 117, 118]. Conversely, MAVS deSUMOylation by SENP1 inhibits its aggregation and antagonizes IRF3 activation [119].

**FIGURE 3:**
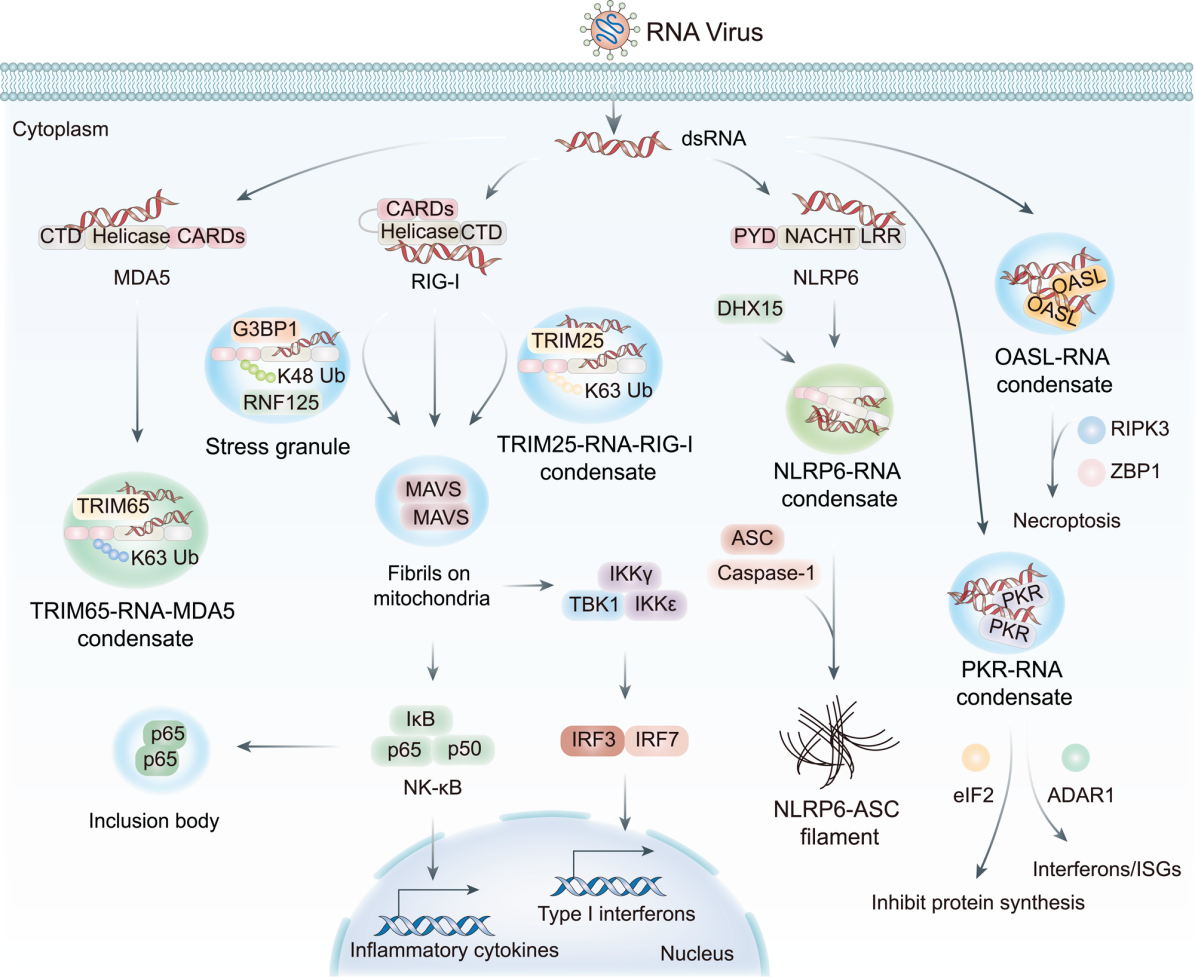
Role of LLPS in innate immunity. E3 ubiquitin ligase, TRIM25, forms liquid‐like condensates with viral RNA. These condensates recruit the RNA sensor RIG‐I and activate it via K63‐linked ubiquitination, consequently initiating downstream signaling through MAVS aggregates. Parallelly, G3BP1 recruits E3 ubiquitin ligase RNF125 to stress granules and destabilizes RNF125. This inhibits the K48‐linked ubiquitination of RIG‐I, which otherwise leads to proteasomal degradation. Additionally, RIG‐I RNA can trigger MAVS fibril formation. However, the p65 subunit of NF‐κB can be trapped in viral replication‐associated inclusion bodies, thereby suppressing proinflammatory cytokine expression. Furthermore, viral RNA drives MDA5 to undergo LLPS. NLRP6 is another pivotal pattern recognition receptor. It forms a liquid‐like NLRP6–dsRNA complex via LLPS that is capable of regulating inflammatory responses. These condensates can recruit and activate the adaptor protein ASC and the effector protein caspase‐1, resulting in filamentous structures that scaffold the NLRP6 inflammasome assembly. OASL scaffolds RIPK3–ZBP1 assembly via phase‐separated liquid droplets, facilitating necroptosis‐mediated antiviral immunity. PKR also undergoes phase separation from dsRNA. This condensate acts as a selective filter, recruiting eIF2 alpha to inhibit protein synthesis and limit virus replication. Moreover, it also recruits ADAR1 and other antiviral factors to enhance immune signaling.

Recent advances reveal that LLPS fundamentally regulates RLR pathway activation. The SG component G3BP1 functions as a regulatory node within this pathway. By recruiting the E3 ligase RNF125 into SGs, G3BP1 attenuates RNF125‐mediated K48‐linked ubiquitination and degradation of RIG‐I, thereby stabilizing the sensor and potentiating signaling (Figure 3) [34, 120, 121, 122]. This intricate regulation demonstrates how MLOs can fine‐tune immune responses through the spatial organization of both positive and negative regulators.

#### 4.1.2 NLRP6 Inflammasomes

The NOD‐like receptor family pyrin domain‐containing 6 (NLRP6) represents a paradigm of LLPS‐mediated pathogen sensing and inflammasome activation. NLRP6's modular architecture, comprising pyrin, NACHT, and leucine‐rich repeat domains, enables sophisticated regulation through phase separation (Figure 3) [123]. Upon dsRNA recognition, NLRP6 undergoes LLPS to form dynamic, liquid‐like complexes that serve as platforms for recruiting the adaptor protein ASC and effector caspase‐1 [124, 125]. This process triggers inflammasome assembly, resulting in the maturation and secretion of interleukin (IL)‐1β and IL‐18, along with induction of pyroptotic cell death, which are all critical responses for controlling viral infections [126].

The formation of NLRP6 inflammasomes is driven by multivalent interactions between IDRs and dsRNA, primarily mediated by electrostatic forces [123, 127]. This process exhibits concentration‐dependent characteristics, ensuring precise activation thresholds and length‐dependent affinity that may facilitate discrimination between viral and host RNA [123]. The dynamic, liquid‐like properties of NLRP6 aggregates enable rapid integration of diverse stimuli and versatile downstream outputs, distinguishing them from more static signaling complexes. The liquid properties of NLRP6 aggregates represent a departure from traditional solid‐like signalosomes, enabling dynamic integration of diverse stimuli and versatile downstream outputs [128, 129]. Notably, NLRP6 aggregates also regulate IFN responses through interactions with RNA helicase DHX15, highlighting its dual functionality in inflammasome activation and antiviral immunity.

#### 4.1.3 PKR and OASL Systems

Additional RNA‐sensing mechanisms leverage LLPS for antiviral defense. The oligoadenylate synthase‐like (OASL) protein undergoes liquid‐like phase transition upon RNA virus stimulation, forming cellular droplets that recruit necroptosis signaling components ZBP1 and RIPK3. This promotes RIPK3 nucleation and signal activation, with OASL1‐deficient mice exhibiting uncontrolled viral dissemination and lethality following infection [130, 131]. Similarly, dsRNA‐dependent PKR undergoes phase separation through multivalent interactions upon viral dsRNA detection, forming condensed structures that function as selective filters. These PKR condensates recruit eukaryotic initiation factor 2 alpha to inhibit protein synthesis and limit viral replication, while also enlisting ADAR1, NLRP1, and DHX9 to further enhance immune signaling (Figure 3) [131, 132, 133].

### 4.2 DNA Sensing Pathways

#### 4.2.1 cGAS–STING–IRF3 Pathway

**FIGURE 4:**
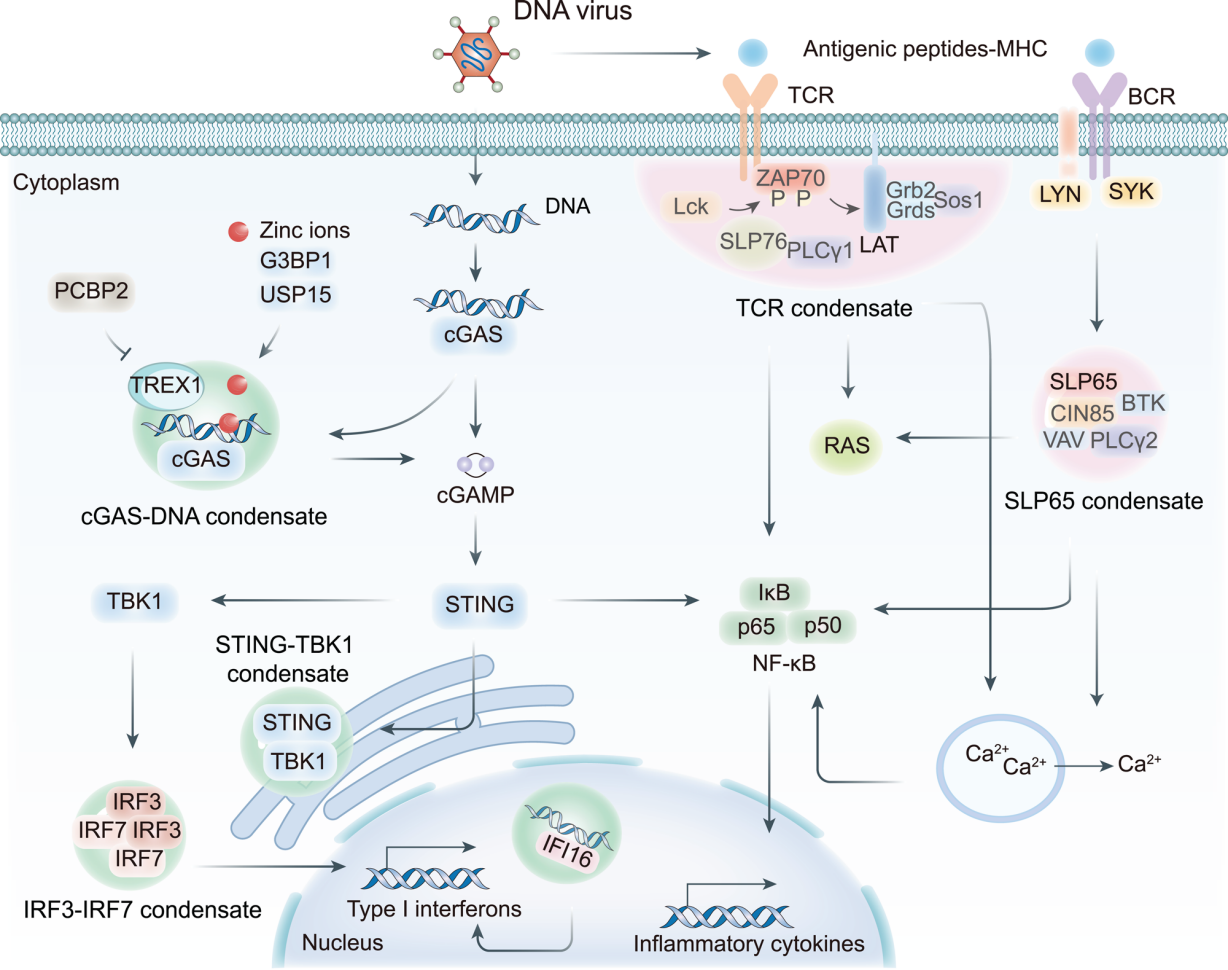
Role of LLPS in innate and adaptive immunity. cGAS forms liquid‐like condensates with dsDNA, which protects it from TREX1‐mediated degradation and enhances cGAMP production. cGAMP subsequently activates STING and TBK1, thereby promoting the formation of IRF3–IRF7 condensates and induction of Type I interferons. Meanwhile, excess cGAMP production triggers LLPS of ER‐resident STING. This results in the recruitment of TBK1 but excludes IRF3 to prevent overactivation of the innate immune response. In the nucleus, IFI16 undergoes LLPS after binding to viral DNA, consequently inducing antiviral gene expression. Adaptive immunity—TCR signaling: LCK phosphorylates the T‐cell receptor (TCR) complex upon engagement of the MHC antigen. The phosphorylated TCR complex recruits the kinase ZAP70, which subsequently phosphorylates LAT. Phosphorylation leads to the formation of liquid‐like LAT condensates. These LAT microclusters accumulate adaptor and effector proteins (such as Grb2, SLP76, Sos1, and PLCγ1) that trigger the activation of downstream pathways, including RAS, calcium influx, and NF‐κB. BCR signaling: in B cells, antigen‐MHC engagement leads to the formation of liquid condensates by the scaffolding protein SLP65 and its binding chaperone CIN85, through multivalent interactions. The proximity of these condensates to the plasma membrane further initiates RAS and NF‐κB signaling.

LLPS plays a central and multilevel regulatory role in the cytoplasmic DNA‐sensing cGAS–STING pathway. DNA binding induces conformational changes in cGAS and robustly drives its condensation into liquid‐like droplets via LLPS. These droplets function as microreactors that concentrate cGAS and its substrates, thereby potently enhancing cGAMP production [9]. This initial LLPS event acts as a crucial signal amplification mechanism, ensuring a robust immune response even to limited DNA stimuli. The process is driven by the N‐terminal IDR of cGAS, rich in positively charged residues, and is enhanced by DNA multivalency, dsDNA length (molecules >45 bp form more stable ladder‐like dimer networks with stronger activity), and zinc ions, which stabilize DNA–cGAS interactions [134, 135, 136, 137]. Regulatory proteins like G3BP1 and USP15 further amplify cGAS condensate formation and subsequent STING signaling through their own disordered regions (Figure 4) [120, 138, 139].

The synthesized cGAMP then activates the endoplasmic reticulum‐resident adapter STING, which itself undergoes LLPS to form aggregates that facilitate its trafficking via the ERGIC [138, 140]. This represents a second strategic use of phase separation, organizing, and coordinating the signaling complex during transport. The kinase TBK1 is recruited as a client protein into these STING condensates, where it is activated and subsequently phosphorylates IRF3. This leads to IRF3 dimerization, nuclear translocation, and ultimate production of IFNs [138, 140, 141, 142, 143].

The system incorporates sophisticated regulatory mechanisms: higher cGAMP concentrations can stimulate a transition of STING condensates into gel‐like states, which sequester STING–TBK1 complexes from IRF3 and prevent overactivation of innate immunity [144]. Downstream, the transcription factor IRF3 undergoes distinct, regulated LLPS in the nucleus upon viral infection, a process that is enhanced by both IFN‐stimulated response elements and IRF7 (Figure 4) [145]. Deacetylation of IRF3/IRF7 by SIRT1 is crucial for this LLPS and IFN gene transactivation, illustrating how PTMs fine‐tune the phase separation of immune signaling components [145, 146].

The potential role of LLPS in regulating nuclear cGAS activity presents an intriguing frontier. While nuclear cGAS is typically tethered to chromatin to prevent aberrant activation by self‐DNA, certain viral infections can disrupt nuclear homeostasis in ways that may promote cGAS condensation. For instance, viral proteins can induce centromeric DNA amplification, releasing self‐DNA fragments that could serve as multivalent scaffolds for cGAS LLPS [147]. Alternatively, nuclear‐invasive viruses can be sensed by host proteins; for example, the protein NONO assembles multimeric complexes specifically upon recognizing the HIV‐2 capsid in the nucleus, potentially creating platforms that recruit and concentrate cGAS [148]. These pathways exemplify how viral intrusion might overcome chromatin‐mediated suppression by generating conditions favorable for phase separation.

In contrast, HIV‐1 co‐opts the principle of phase separation for immune evasion. It forms nuclear MLOs that sequester viral DNA, physically shielding it from cGAS and other nuclear sensors [149]. Pharmacological disruption of these condensates using agents such as lenacapavir and PF74 exposes the viral DNA and triggers a cGAS‐mediated innate immune response [150]. This evasion strategy is further supported by the virus's ability to recruit and inactivate cGAS via its unintegrated DNA's chromatin‐like structure and by usurping host factors like CPSF6, whose phase separation facilitates viral capsid entry [149, 151].

The molecular architecture of cGAS provides a plausible foundation for its engagement in phase separation within the nucleus. The cGAS N‐terminal IDR, known to drive cytoplasmic LLPS, also mediates nuclear chromatin interactions. Phosphorylation of this domain during mitosis concurrently disrupts both DNA binding and phase separation capacity, suggesting a conserved regulatory module [152]. Moreover, an additional DNA‐binding interface within the catalytic domain enhances both enzymatic activity and liquid‐phase condensation [135, 153]. These shared features support the hypothesis that the mechanisms governing cGAS condensation could be operational in the nucleus.

In conclusion, while direct evidence for nuclear cGAS LLPS remains to be established, the conceptual framework of phase separation offers a powerful mechanistic lens. It can explain how nuclear cGAS might be liberated from chromatin for activation and how its function can be effectively subverted. The regulation of nuclear cGAS levels, influenced by the cell cycle and other signals, adds further complexity. Investigating the nuanced roles of LLPS in this compartment is therefore a critical frontier for understanding antiviral immunity and autoinflammatory diseases.

#### 4.2.2 IFI16 DNA Sensing

IFN‐inducible protein 16 (IFI16) represents another crucial DNA sensor that leverages LLPS for antiviral function. As a PYHIN family member, IFI16 contains HIN200 domains for DNA binding and a pyrin domain for oligomerization. Upon viral DNA binding, IFI16 undergoes LLPS to form dynamic IFI16‐DNA droplets, which then undergo phosphorylation‐dependent stabilization and transformation into solid filaments (Figure 4) [154, 155, 156, 157, 158, 159]. This phase transition marks the shift from sensing to active signaling, with these solid filaments serving as scaffolds for downstream signaling complex assembly [9, 158, 160].

IFI16 condensation enhances antiviral function through multiple mechanisms: clustering increases local concentration for improved detection sensitivity and accelerated signal transduction; dynamic properties enable rapid adaptation to environmental changes; and interactions with STING and other signaling proteins facilitate the assembly of a large signaling complex [158, 161, 162]. The formation of stable solid filaments ensures signaling persistence, enabling sustained immune activation against viral threats [154, 162].

### 4.3 Adaptive Immunity

#### 4.3.1 T‐Cell Receptor Signaling

T‐cell receptors (TCRs) initiate adaptive immune responses through antigen recognition, with LLPS playing crucial roles in signal amplification and integration. TCR engagement triggers formation of dynamic membrane microclusters through IDR‐mediated phase separation of LAT and its binding partners [34, 163]. These LAT condensates function as platforms for amplifying and integrating TCR‐mediated signals, recruiting key signaling molecules including GRB2, SOS1, and PLCγ1 through multivalent interactions (Figure 4) [164, 165, 166, 167, 168, 169, 170].

This LLPS‐driven organization enhances signal specificity and amplitude, enabling precise T cell activation, proliferation, and differentiation. The material properties of these condensates, ranging from liquid to more gel‐like states, can influence signal duration and strength, adding another layer of regulation to T cell responses. Viruses have evolved sophisticated mechanisms to disrupt TCR LLPS. The HIV‐1 Nef protein directly binds to TCR proline‐rich regions, blocking phase separation‐dependent SLP76–NCK1 interactions and causing T cell migration defects that promote viral latency [171]. The HCV core protein and NS3/4A protease inhibit LAT phosphorylation and IDR‐mediated phase separation, impairing viral clearance [44, 172, 173]. Additionally, the influenza NS1 protein may interfere with TCR signaling LLPS through LAT binding, potentially contributing to immune imbalance and cytokine storm development [174, 175].

#### 4.3.2 BCR Signaling

BCR activation similarly employs LLPS for signal transduction, with adaptor protein SLP65 (BLNK) undergoing phase separation through multivalent interactions between its proline‐rich domain and CIN85 SH3 domains (Figure 4) [176, 177, 178, 179]. SLP65 condensates trigger downstream pathways including RAS and NF‐κB, affecting B cell proliferation, differentiation, and antibody production, which are essential processes for humoral immunity [180, 181, 182]. The formation of these signaling condensates is tightly regulated, with their composition and material properties influencing the signal outcome.

Viral interference targets BCR LLPS through multiple strategies. The HIV‐1 Nef binds to SLP65 proline‐rich domains, blocking CIN85 interactions and inhibiting phase separation to reduce antibody secretion [171]. The influenza NS1 protein inhibits E3 ligase CBL degradation, prolonging BCR signaling and exacerbating inflammatory damage [183]. The Epstein–Barr virus LMP2A protein mimics BCR signaling, hijacking SLP65 condensate localization to promote B cell immortalization [184]. These examples highlight the critical importance of LLPS in adaptive immunity and the evolutionary pressure on viruses to develop countermeasures.

## 5 Viral Subversion of LLPS for Immune Evasion

The evolutionary arms race between viruses and hosts has driven the development of sophisticated viral strategies to subvert LLPS‐mediated immune defenses. Viruses do not merely evade these defenses; they actively reshape the biophysical landscape of the host cell, co‐opting the principles of phase separation to create “immune‐privileged” microenvironments that favor replication while minimizing detection [185, 186]. This section synthesizes the diverse mechanisms by which different viral families interfere with, inhibit, or repurpose host LLPS processes, revealing both convergent themes and unique adaptations.

### 5.1 Spatial Sequestration and Insulation within Viral Condensates

A primary strategy employed by diverse viruses is the spatial sequestration of both viral components and host immune factors within LLPS‐driven condensates, effectively insulating them from the antiviral machinery of the cell.

#### 5.1.1 Positive‐Sense RNA Viruses: SARS‐CoV‐2

**FIGURE 5:**
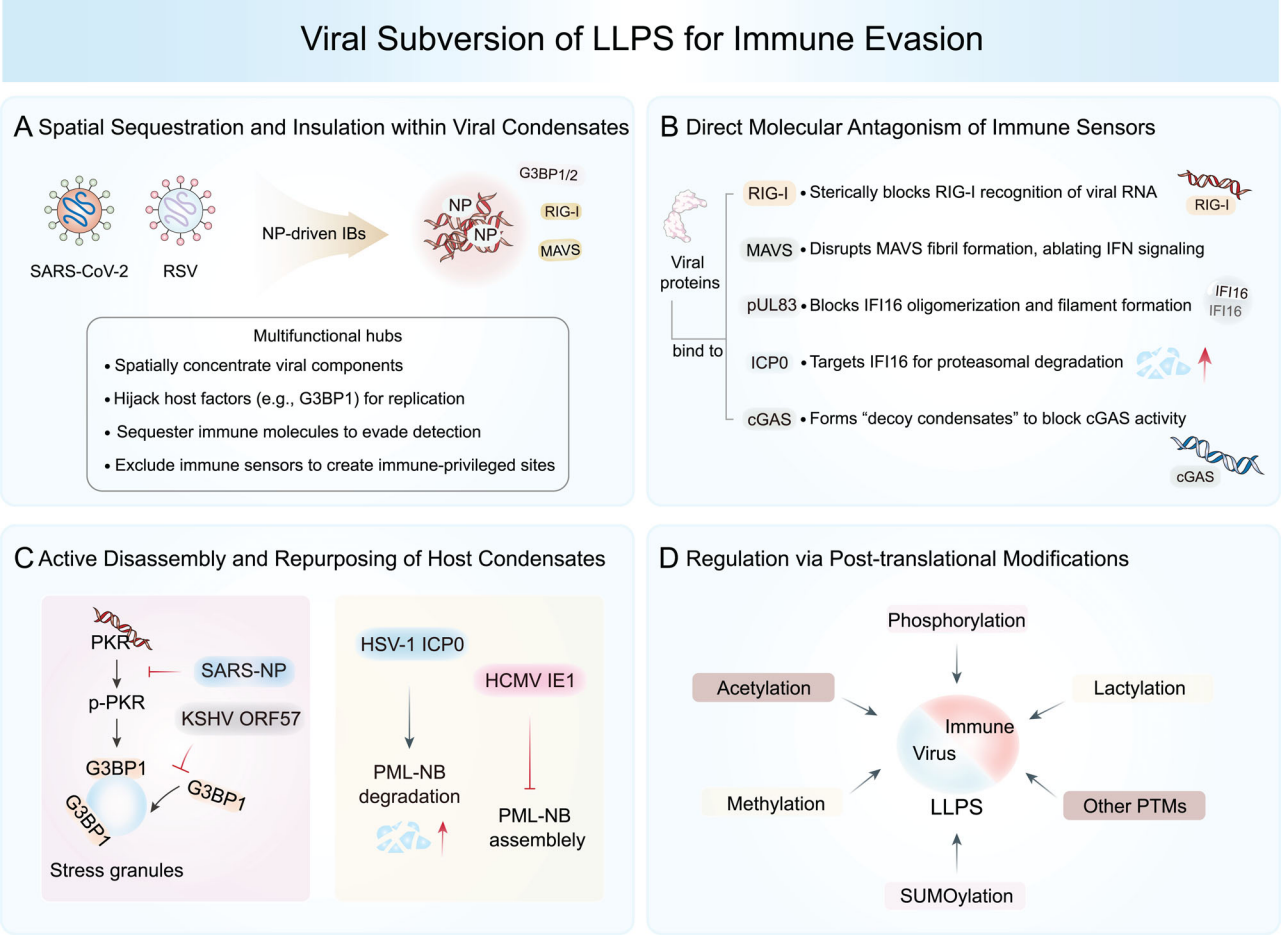
Pathogens utilize biomolecular condensates to escape innate immunity. (A) Viral inclusion bodies (IBs) function as multifunctional hubs that spatially concentrate viral RNA and replication machinery to evade immune sensors such as RIG‐I and MDA5, while simultaneously excluding or sequestering key components of antiviral response, such as p65, MAVS, and SGs. Viral components can directly bind to and inhibit the core immune‐sensing pathways, often by exploiting or disrupting their LLPS capacity. For example: (B) SARS‐NP binds RIG‐I to hinder viral RNA recognition, while KSHV ORF52, HSV‐1 VP22, and VZV ORF9 interact with cGAS to suppress its activity. (C) Disrupting LLPS capacity of core immune sensors is also another crucial evasion strategy. HSV‐1 ICP0 and HCMV pUL83 inhibit IFI16 phase separation and promote its degradation. (D) Viruses can also exploit LLPS to modulate immune signaling. KSHV ORF33 undergoes condensation with STING/MAVS or PPM1G, thereby inhibiting IRF3 recruitment and activation. (E) Viral components interfere with the phase separation of host immune molecules. KSHV ORF57 impairs stress granule assembly by disrupting PKR binding and autophosphorylation. (F) Viral proteins disrupt PML‐NBs through distinct mechanisms. HSV‐1 ICP0 promotes PML‐NB degradation, while HCMV IE1 inhibits de novo SUMOylation required for PML‐NB integrity and assembly.

SARS‐CoV‐2 exemplifies this strategy through its NP, which drives the formation of dynamic IBs. These IBs function as multifunctional hubs that spatially concentrate viral RNA and replication machinery, while simultaneously excluding or sequestering key innate immune sensors [187, 188]. By creating a physical barrier, these condensates reduce the accessibility of viral pathogen‐associated molecular patterns, such as genomic RNA, to cytosolic sensors like RIG‐I and MDA5, thereby blunting the initiation of IFN responses [53, 189, 190, 191]. Beyond passive exclusion, SARS‐NP actively recruits and traps host factors like DDX3X and G3BP1/2, repurposing them for viral replication while disrupting the assembly of antiviral SGs (Figure 5A) [3, 117, 192, 193, 194, 195, 196].

#### 5.1.2 Negative‐Sense RNA Viruses: RSV

Respiratory syncytial virus (RSV) utilizes a similar sequestration strategy but with a broader scope. Its IBs act as molecular sinks, entrapping a wide array of critical immune signaling molecules [197, 198]. Key components of the antiviral response, including MAVS, MDA5, the NF‐κB p65 subunit, and p38 MAPK, are selectively concentrated within these liquid organelles through interactions with the viral NP (Figure 5A) [14, 199, 200]. This sequestration disrupts the formation of functional signaling complexes, prevents nuclear translocation of transcription factors, and effectively neutralizes multiple signaling axes of the innate immune system.

### 5.2 Direct Molecular Antagonism of Immune Sensors

Viruses have evolved proteins that directly bind to and inhibit the core components of immune‐sensing pathways, often by exploiting or disrupting their LLPS capacity.

#### 5.2.1 Targeting RNA Sensing: SARS‐CoV‐2

Viruses achieve robust immune suppression through direct molecular interference. For example, the SARS‐NP binds directly to RIG‐I, sterically preventing its recognition of viral RNA (Figure 5B) [193]. It also disrupts the multiubiquitination and subsequent prion‐like aggregation of MAVS, a critical step for downstream IRF3 activation and IFN production [117, 192]. Moreover, other viruses such as HCMV and HSV‐1 target the DNA‐sensing pathway: HCMV pUL83 inhibits IFI16 oligomerization, while HSV‐1 ICP0 promotes IFI16 degradation (Figure 5C) [156, 201]. This multitarget strategy ensures robust suppression of the initial wave of antiviral signaling.

#### 5.2.2 Targeting DNA Sensing: Herpesviridae

Herpesviruses employ multiple strategies to subvert cGAS–STING signaling. Viral proteins such as KSHV ORF52, HSV‐1 VP22, and VZV ORF9 function as molecular decoys by binding directly to cGAS or DNA, thereby inhibiting their interaction. Notably, these viral proteins undergo DNA‐induced LLPS, forming “decoy condensates” that lack enzymatic activity and effectively compete with cGAS for cytosolic DNA, suppressing cGAMP production (Figure 5B) [202, 203, 204]. KSHV adds another layer of regulation through ORF33, which undergoes phase separation with both STING and MAVS. This interaction inhibits IRF3 recruitment and recruits the host phosphatase PPM1G, which dephosphorylates and inactivates STING and MAVS, further suppressing their condensation and activation (Figure 5D) [205]. The functional importance of this mechanism is underscored by the enhanced IFN‐β production observed upon ORF33 deletion [205].

### 5.3 Active Disassembly and Repurposing of Host Condensates

Instead of simply evading host condensates, some viruses actively induce their disassembly or hijack their components.

#### 5.3.1 Subversion of SGs

SARS‐NP competitively binds the core SG nucleator G3BP1, effectively dissolving preformed SGs and repurposing their components (e.g., hnRNPs, G3BP1/2) for viral replication. This hijacking enhances viral RNA stability and ensures continued protein synthesis, with G3BP1 knockout significantly reducing viral replication in lung organoids [97, 206, 207, 208]. KSHV ORF57 inhibits the SG component PKR by disrupting its dsRNA binding and autophosphorylation, thereby impairing SG assembly (Figure 5E) [209].

#### 5.3.2 Disruption of Nuclear Bodies

Herpesviruses directly target nuclear MLOs. HSV‐1 ICP0 promotes the degradation of PML nuclear bodies (PML‐NBs), key structures involved in intrinsic antiviral defense [156, 201]. Proteomic analysis has identified numerous HSV‐1 proteins with high LLPS potential participating in critical viral processes and host interactions [210]. Similarly, HCMV IE1 inhibits the de novo SUMOylation required for PML‐NB integrity and function (Figure 5F). These actions constitute a targeted inhibition of LLPS‐dependent nuclear antiviral structures [211, 212, 213].

### 5.4 Regulation via PTMs

Viruses and hosts engage in a tug‐of‐war over the PTM status of viral scaffold proteins, which directly influences LLPS and immune evasion. For SARS‐NP, SUMOylation at Lys65, mediated by the host E3 ligase TRIM28, enhances its oligomerization and phase separation, thereby promoting viral replication. Conversely, acetylation at Lys375 inhibits NP LLPS and diminishes its ability to disrupt MAVS signaling [214]. This dynamic regulation of viral scaffold PTMs represents a critical virus–host interface and highlights potential therapeutic strategies aimed at shifting the balance toward inhibitory modifications.

In summary, viral subversion of LLPS is a multifaceted and highly effective immune evasion strategy. It encompasses spatial insulation via condensate formation, direct molecular antagonism of immune sensors, active disassembly or repurposing of antiviral condensates, and sophisticated manipulation of PTM networks. Although current research has illuminated many of these pathways, a comprehensive understanding of the rules governing the specificity of recruitment and exclusion across different viral families and infection stages remains an open frontier. Future investigations should focus on the bidirectional regulation of LLPS, examining how host factors may in turn disrupt viral condensates, and extend these studies to emerging viruses to fully exploit this battlefield for therapeutic gain.

## 6 Therapeutic Frontiers: Targeting the Phase Separation Interface

**FIGURE 6:**
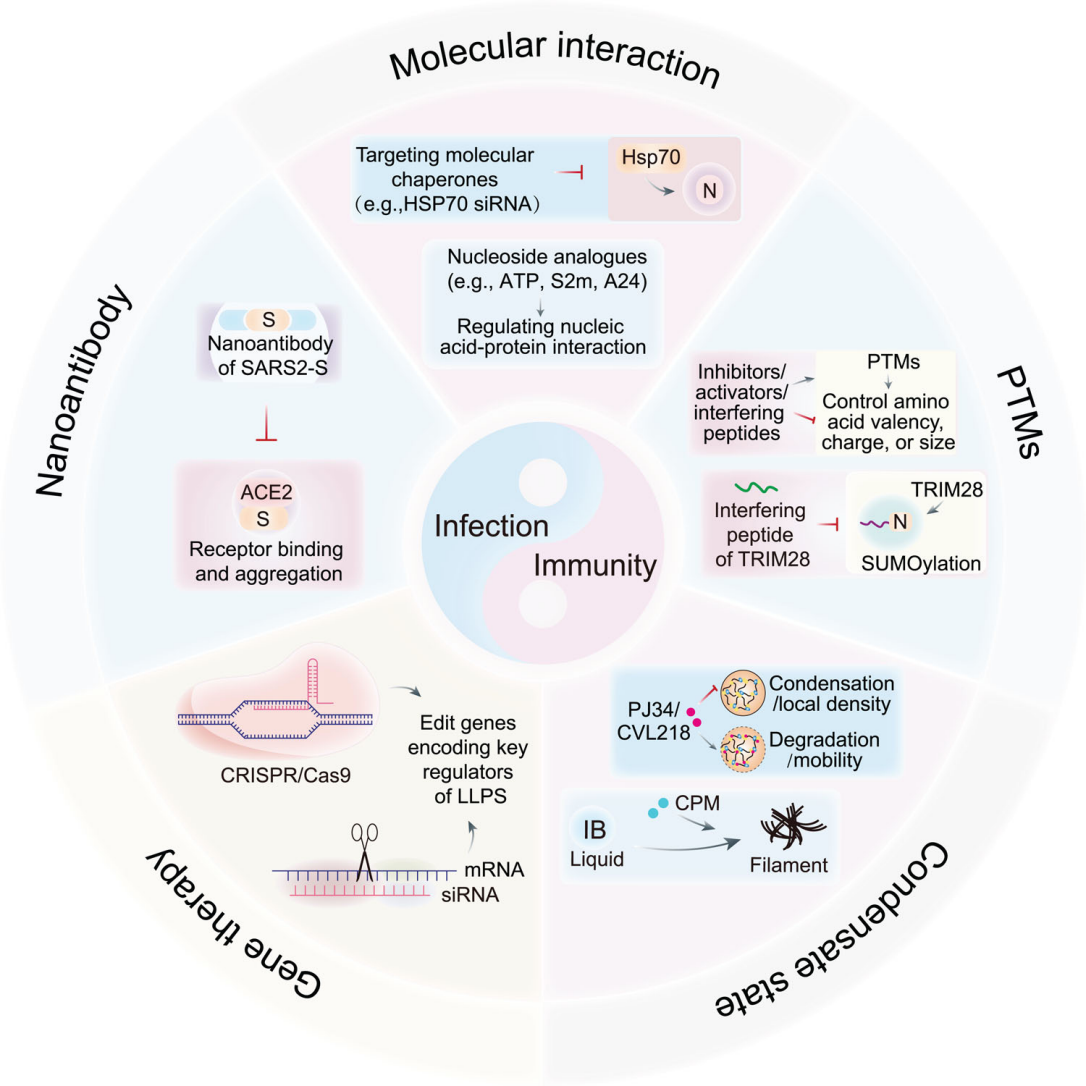
Future prospects of viral infection therapy via LLPS regulation. Future antiviral strategies could exploit LLPS regulation by utilizing multiple approaches. Targeting molecular interactions: targeting molecular chaperones with small molecules or siRNA can disrupt or enhance condensation between molecules (e.g., siRNA‐mediated silencing of Hsp70 blocks SARS‐CoV‐2 nucleocapsid–RNA condensation). Modifying posttranslational modifications (PTMs): inhibitor/activator/interfering peptides of scaffold proteins can directly impact the multivalent interactions between macromolecules by altering their valency, charge, and size (e.g., TRIM28‐targeting peptides suppress nucleocapsid SUMOylation and condensate formation). Altering condensate biophysical states: small molecules, such as PJ34/CVL218, decrease the local density of condensates and increase the permeability, whereas cyclopamine (CPM) forces liquid‐to‐solid transition of viral inclusion bodies (IBs; e.g., in respiratory syncytial virus), thereby impairing replication efficiency. Editing LLPS machinery: CRISPR/Cas9 or siRNA‐targeting scaffold proteins (e.g., RNA‐binding proteins, chaperones) precisely modulate phase separation initiation. Blocking viral entry: engineered nanoantibodies against SARS‐CoV‐2 spike protein (S) prevent ACE2‐mediated membrane fusion and condensate formation at entry sites.

The pervasive role of biomolecular condensates in viral replication and host immunity presents a fundamentally new therapeutic paradigm [215]. Unlike traditional targets (e.g., viral enzymes), LLPS‐based therapies exploit the biophysical rules of condensate formation, targeting multivalent interactions, physicochemical properties, or regulatory networks, to disrupt pathogenic condensates while preserving essential host functions (Table 1) (Figure 6). However, the shared reliance of viruses and hosts on LLPS creates a “double‐edged sword”: inhibiting host LLPS to block viral replication may compromise immune defenses, whereas enhancing immune condensates could provoke excessive inflammation. Resolving this tension requires strategies that prioritize virus‐specific condensate features and spatiotemporal precision. In the sections below, we systematically dissect four interrelated therapeutic frontiers, integrating mechanistic insights, preclinical evidence, and translational challenges to provide a comprehensive framework for targeting LLPS in viral infections.

**TABLE 1:** Antiviral therapy targeting phase separation.

Strategy	Target	Mechanism	Representative case/molecule	References
Molecular chaperones	Hsp70 inhibitor	Small molecules (e.g., JG40 and quercetin) bind to Hsp70's ATPase domain, thereby inducing LLPS of viral IBs and blocking virus replication and inflammatory factor release.	JG40, quercetin	[216, 217, 218, 219]
	GRP78 inhibitor	Downregulating GRP78 via siRNA or HA15 inhibits membrane fusion, viral entry (e.g., SARS‐CoV‐2), and protein synthesis.	HA15, GRP78 siRNA	[220, 221, 222]
Stress granules	CK2 inhibitor	Restores SG antiviral function by inhibiting the abnormal interaction between viral N protein and SG core protein (e.g., G3BP1/2). Silmitasertib reverses the innate immune response by blocking CK2‐mediated hyperphosphorylation of SG protein.	Silmitasertib, GO289	[223, 224]
Nucleoside analogues	Nucleic acid	ATP binds to nucleic acids or arginine residues in the proteins, competitively dislodging the nucleic acids from the proteins and solubilizing the LLPS.	ATP	[225]
	Multivalent nucleic acid aptamer	Neutralizes the nucleocapsid protein IDR charge balance through multivalent electrostatic interactions (e.g., positively charged S2m and A24), dissolving viral RNP aggregates.	S2m, A24	[225]
	Cyclic bivalent adapter	Circular DNA aptamers (e.g., cb‐N‐Apt17) anchor NP dimer SR domains, reducing the dissociation constant to the pM level and resisting virus escape mutations.	cb‐N‐Apt17	[226]
SUMOylation regulation	Interfering peptide	Blocks TRIM28‐mediated SARS2‐NP SUMOylation, inhibiting its oligomerization, RNA binding, and LLPS to suppress virus replication and restore innate immunity.	—	[196]
Acetylation/deacetylation regulation	HDAC6 inhibitor	Inhibits HDAC6, modulating DDX3X acetylation and SG formation	Tubacin	[227, 228]
	p300/CBP inhibitor	Suppresses acetyltransferase activity, impairing DDX3X acetylation and SG assembly	A‐485, C646	[227, 228]
	HDAC inhibitor	Promote acetylation of SARS‐NP, inhibit its LLPS and viral replication	Trichostatin A	[229]
	SIRT1 activator	Enhances IRF3 deacetylation, restoring LLPS and IFN‐I expression for antiviral immunity	Resveratrol, SRT2183	[230, 231]
Phosphorylation regulation	SRPK1 activator	Phosphorylates SARS‐NP SR region, weakening RNA‐induced LLPS and viral transcription	—	[232]
Methylation regulation	PRMT inhibitor	Inhibits arginine methylation, disrupting LLPS of FUS/SARS‐CoV‐2 and viral–host complexes	GSK3368715	[233, 234, 235]
Destruction of condensed matter	Hydrophobic disruptor	Nonspecific interference with hydrophobic/hydrophilic interactions within the condensed body, leading to droplet dissolution.	1,6‐Hexanediol	[236]
	Solvent substitute	As a nontoxic substitute for 1,6‐hexanediol, it completely dissolves the rotavirus replication plant (in the early‐stage of infection), but is ineffective for late‐stage infected aggregates.	Propylene glycol	[136]
	SARS‐NP/RNA interaction	GCG binds SARS‐NP, blocking RNA binding and LLPS.	Gallocatechin gallate (GCG)	[237]
	Interaction of SARS‐NP and RNA	Interference virus LLPS through RNA binding; its structure can be optimized to form broad‐spectrum antiviral analogs (e.g., conformationally restricted aminoglycosides).	Kanamycin	[238]
	Interaction of SARS‐NP and RNA	Blocking the binding of SARS‐NP NTD domain to RNA, inhibiting the formation of RNP complexes and virus lifecycle	Ceftriaxone sodium	[239]
	Interaction of SARS‐NP and RNA	Targeting both SARS‐NP NTD and CTD domains to inhibit nucleic acid binding and disrupt LLPS, while the HCQ–CTD complex structure serves as a drug optimization template.	Hydroxychloroquine, HCQ	[240]
CRISPR/Cas9	PKR	PKR knockout disrupts viral hijacking of host antiviral factors (e.g., cyclophilin A–PKR interaction), suppressing replication.	—	[241, 242]
Small nucleic acid	miRNA‐122	Nucleic acid drugs can effectively reduce the expression level of pathogenic proteins by precisely targeting specific mRNA.	Miravirsen	[243, 244]
Protein editing technology	LCB1–PNGase	Fusing targeted peptides (e.g., LCB1) with deglycosylation enzymes (PNGase) directly edits N‐glycosylated proteins in live cells, removes glycosylation of SARS‐CoV‐2 spike protein, and changes asparagine residues to aspartic acid.	LCB1–PNGase	[245, 246]
Hardened condensed matter	RSV M2‐1	Inducing liquid–solid phase transformation of IBs in RSV‐infected cells, reducing the fluidity of aggregates, and inhibiting virus replication (directly targeting M2‐1 protein)	Cyclopamine, CPM	[247]
	RSV M2‐1	Similar to CPM, inhibiting RSV replication through hardened aggregates	A3E	[247]
	IAV nucleoprotein	Inducing IAV nucleoprotein aggregation, hindering nuclear accumulation, and altering conformation; inhibit virus replication in the body	Nucleozin and its analogues	[248, 249]
	HIV‐1 reverse transcriptase	Form high‐density, low activity HIV‐1 reverse transcriptase aggregates to inhibit viral replication	Efavirenz (EFV)	[250, 251]
Dissolve condensed matter	SARS‐NP	PJ34 and CVL218 alter the morphology and kinetics of SARS‐CoV‐2 NP–RNA–nsp12 complex aggregates, reducing local density and promoting decomposition.	PJ34, CVL218	[252, 253]
Enhance condensed matter	STING	Promote the formation of STING aggregates, prolong the activation time of innate immune pathways, and enhance antiviral immunity	PC7A	[254]
	MERS‐CoV NP	Targeting MERS‐CoV nucleocapsid protein, inducing abnormal aggregation, and inhibiting virus replication	P3 (5‐benzyloxygramine)	[255]
Nanoantibody	SARS‐CoV‐2 neutralizing nanobody	Blocks viral ACE2 binding via steric hindrance/conformational locking, inhibiting invasion; some nanobodies additionally destabilize prefusion spike.	VHH72, Nb6	[256, 257, 258]
Interference peptide	SARS‐NP interference peptide	Synthetic peptides (e.g., d‐amino acid modified peptides) competitively bind to disrupt NPs dimerization, inhibit RNA‐LLPS, restore MAVS K63 ubiquitination/aggregation, and activate IFN‐I pathway.	DD targeted peptide	[229]

### 6.1 Targeting Core Drivers: Disrupting Multivalent Interactions

The most straightforward strategy is to target the multivalent interactions that scaffold viral condensates. This can be achieved by targeting chaperones, SG regulators, or nucleic acid scaffolds, with minimal off‐target effects if interventions are tailored to virus‐specific interactions.

Molecular chaperones (e.g., Hsp27, Hsp40, Hsp70, Hsp90) regulate LLPS by modulating protein folding, oligomerization, and solubility—functions co‐opted by viruses to stabilize viral condensates [216, 259, 260, 261].

The therapeutic promise of this approach is exemplified by Hsp70 inhibition. In RABV and RSV infections, Hsp70 is recruited to viral IBs, where it interacts with viral NPs and polymerases to enhance their stability and enzymatic activity. Genetic silencing of Hsp70 via small interfering RNA (siRNA) or inhibiting it with quercetin abolishes RABV transcription, translation, and progeny production, validating Hsp70 as a critical cofactor for viral condensate function [217, 218, 219]. More specific and potent small‐molecule Hsp70 inhibitors (e.g., JG40) exhibit broad‐spectrum activity against flaviviruses (e.g., DENV), destabilizing NS3/NS5 polymerase condensates while simultaneously suppressing proinflammatory cytokine production—addressing both viral replication and immunopathology [220]. Similarly, cell surface glucose‐regulated protein 78 (GRP78) is hijacked by SARS‐CoV‐2 to facilitate viral entry and replication [221, 262, 263]. Ablation of GRP78 expression using siRNA or HA15 not only hinders the entry of several viruses but also reduces the production of viral proteins in host cells and mice [222, 262, 264]. However, the “double‐edged sword” nature of this strategy is evident: Hsp90 and its cochaperone SGT1 are essential for the stability of NLR family pathogen sensors, which assemble into immune condensates to initiate inflammatory responses. Inhibiting Hsp90 reduces NLR protein levels, compromising antipathogen immunity [223]. This highlights the need for chaperone inhibitors with cell‐type or condensate‐specific activity, potentially achievable through tissue‐targeted delivery systems (e.g., lipid nanoparticles, LNPs, for lung epithelial cells) to mitigate systemic cytotoxicity.

Viruses frequently evade antiviral responses by disrupting SGs. For instance, NPs interact with the SG proteins G3BP1/2 and CSNK2B/CSNK2A2, the subunits of CK2, to disassemble or inhibit SG formation. Thus, small molecules that disrupt the protein–protein interactions between viral NPs and SG‐associated proteins could serve as potential SG‐targeting chemical probes or antivirals; examples include CK2 inhibitors such as GO289 and silmitasertib [224, 225]. However, the therapeutic window for SG modulation may be narrow. Excessive SG activation may trigger a storm of inflammatory factors, and reversible regulatory strategies (such as photocontrolled kinase inhibitors) need to be designed to precisely control the spatiotemporal dynamics of SG.

Nucleoside analogs are a class of compounds that mimic the structure and function of nucleotides. Certain nucleoside analogues have been shown to disrupt the interaction between nuclear proteins and RNA, thereby inhibiting LLPS formation. ATP can competitively bind specifically to the RBD of NPs, thereby modulating LLPS of the SARS‐NP. ATP binds to nucleic acids as well as to arginine residues in the proteins, competitively dislodging the nucleic acids from the proteins and solubilizing the LLPS. S2m (derived from the 32‐polymer stem‐loop II of the SARS‐CoV‐2) and A24 (a 24‐oligomer nonspecific nucleic acid) can modulate LLPS through dynamic and multivalent interactions with arginine/lysine residues in the structural domains and disordered regions of proteins [226]. Moreover, a single‐stranded DNA aptamer (N‐Apt17) and its circular bivalent form (cb‐N‐Apt17) can effectively disrupt the LLPS of the SARS‐NP, which presents a potential inhibitory effect on viral replication [265].

Targeting condensates can disrupt viral replication centers while simultaneously affecting host stress responses, exemplifying the “double‐edged sword” of LLPS‐directed interventions. Consequently, next‐generation inhibitors should focus on virus‐specific interaction interfaces, such as developing small molecules targeting viral protein–host chaperone protein complexes through structure‐based drug design, rather than broad‐spectrum chaperone protein inhibitors. A more refined strategy involves allosteric modulation of condensates instead of dissolving them; small molecules or peptides could bind key scaffold proteins to subtly alter their valency or conformation, thereby shifting the phase boundary and modifying condensate composition and function. This approach provides a more precise therapeutic window by modulating, rather than eliminating, condensate activity.

### 6.2 Regulating the Physicochemical Environment: PTMs and Condensate State

While targeting core drivers aims to dismantle the condensate architecture, a more sophisticated approach seeks to reprogram its functional output by manipulating the physicochemical rules that govern its assembly and material state. This is primarily achieved through two levers: PTMs and direct chemical modulation [1, 8, 137, 266, 267].

PTMs act as a dynamic molecular code, rewriting the charge, hydrophobicity, and valency of scaffold proteins to instruct condensate fate, with SUMOylation serving as a prime example of context‐dependent regulation across viral types. For SARS‐CoV‐2, the SUMO E3 ligase TRIM28 catalyzes SUMOylation of the SARS‐NP, a process essential for SARS‐NP homo‐oligomerization, RNA binding, and condensate formation—processes critical for viral genome packaging and replication. Interfering peptides that block the TRIM28–SARS2‐NP interaction inhibit NP SUMOylation and LLPS, reducing SARS‐CoV‐2 replication while restoring innate antiviral immunity [214]. In contrast, HSV‐1 infection induces global loss of SUMOylated host proteins, driven in part by the viral ICP0 protein; ICP0‐mediated degradation of SUMOylated targets like PML‐NBs impairs IFN‐dependent defenses, lowering HSV‐1 sensitivity to IFN. In contrast, nuclear‐replicating RNA viruses such as IAV upregulate SUMOylation in a manner dependent on active viral RNA polymerases, indicating that SUMO pathways are co‐opted to support nuclear viral replication. This context dependence underscores the therapeutic potential of targeting SUMO machinery: viruses that rely on SUMOylation (e.g., SARS‐CoV‐2, IAV) can be inhibited by disrupting host SUMO enzymes such as TRIM28, with minimal risk of broad cytotoxicity [233].

Acetylation and deacetylation further balance viral replication and immune activation by modifying protein charge and hydrophobicity. For coronaviruses, acetylation of SARS‐NP at Lys375—a residue conserved across SARS‐CoV and Middle East respiratory syndrome coronavirus (MERS‐CoV), adjacent to the NP dimerization domain—abrogates NP LLPS and inhibits viral replication, suggesting HDAC inhibitors (e.g., trichostatin A) could enhance NP acetylation to suppress coronavirus replication [268]. Acetylation also impacts host proteins: acetylation of the RNA‐binding protein TDP‐43 promotes its condensation, and TDP‐43 then colocalizes with SARS‐NP in condensates, impairing TDP‐43‐mediated RNA processing and potentially hijacking host RNA metabolism for viral benefit [227, 269]. In SGs, the component DDX3X is acetylated by CBP and deacetylated by HDAC6. Modulating this balance with drugs, such as the HDAC6 inhibitor tubacin or the CBP inhibitor A‐485, alters SG LLPS and assembly, thereby limiting viral hijacking of these condensates [228, 229]. For immune regulation, deacetylation of the transcription factor IRF3 by SIRT1 is required for IRF3 to form condensates and induce IFN expression. In aged mice, declining SIRT1 activity compromises IRF3 condensates and IFN signaling, but SIRT1 agonists (e.g., resveratrol, SRT501, SRT2183) restore these functions to antagonize viral replication, offering a therapy for age‐related susceptibility to viral infections. Conversely, SIRT1 inhibitors (e.g., EX527) suppress IRF3‐mediated IFN production, providing a strategy for autoimmune diseases driven by excessive IFN signaling [145, 232].

Phosphorylation and arginine methylation also contribute to condensate regulation. The host kinase SRPK1 phosphorylates the SR region of SARS‐NP, reducing the ability of NP to undergo RNA‐induced LLPS and inhibiting viral RNA transcription, making SRPK1 activators potential antivirals to disrupt NP condensates [234]. Lysophosphatidic acid maintains innate antiviral immunity in a proactive state via serine/threonine‐protein kinase 38‐like‐mediated IRF3 Ser303 phosphorylation, which ensures the rapid immune response against the virus [270]. Arginine methylation regulates the LLPS of RBPs such as FUS, which colocalizes with SARS‐NP in condensates and may contribute to NP mRNA processing. Inhibitors of protein methyltransferases (e.g., GSK3368715) can alter FUS arginine methylation, reshaping the composition and biophysical properties of FUS–NP condensates to disrupt viral RNA metabolism [235, 269, 271]. Across different viral systems, PTMs exhibit highly context‐dependent regulation of phase separation. The same modification can have opposite effects depending on the virus or the cellular environment. This underscores the importance of understanding PTMs within the specific virus–host cell context; generalizing conclusions without considering this ternary relationship can be misleading or even counterproductive.

Complementing PTMs, small molecules can directly modulate the material properties of condensates, providing rapid pharmacological intervention. To dissolve viral condensates, 1,6‐hexanediol is widely used to disrupt hydrophobic and hydrophilic interactions, but its application is limited by high cytotoxicity (5–10%, v/v) and off‐target inhibition of kinases and phosphatases [136, 236]. Propylene glycol—a nontoxic alternative—effectively dissolves rotavirus RFs, although late‐stage rotavirus infection alters NSP5–NSP2 condensate properties to confer resistance [237]. The development from nonspecific agents like 1,6‐hexanediol to more specific compounds such as gallocatechin gallate (GCG) illustrates the evolution from chemical probes to lead therapeutics.

Targeted disruptors include (–)‐GCG, a polyphenol that binds SARS‐NP at noncytotoxic doses to reduce RNA‐binding affinity and block RNA‐induced LLPS, suppressing replication [238, 272]. However, most current molecules cannot distinguish between viral and host condensates. Future efforts should leverage biophysical and structural biology approaches to rationally design compounds that specifically recognize the unique molecular signatures of viral proteins. Aminoglycosides, such as kanamycin disrupt LLPS in SARS‐CoV‐2‐ or HIV‐infected cells by binding viral RNA [239]; their structural adaptability allows design of conformationally constrained analogs for specific targeting of viral condensates [240]. Ceftriaxone sodium blocks RNA binding to the NTD of SARS‐CoV‐2 to prevent NP–RNA RNP condensate formation, while hydroxychloroquine (HCQ) binds the NTD and CTD to inhibit NP–nucleic acid interactions and LLPS with the HCQ–CTD complex structure guiding development of high‐affinity NP‐specific drugs [247, 273]. Additionally, PJ34 and CVL218 act as “condensate expanders,” binding SARS‐NP to reduce condensate density, increase permeability, and drive disassembly of NC–RNA–nsp12 condensates [253, 254].

Inducing liquid‐to‐solid transitions of viral condensates restricts mobility to suppress replication. For RSV, cyclopamine (CPM)—a sonic hedgehog pathway antagonist—and its inactive analog A3E trigger rapid IB hardening in infected cells; resistance arises from an R151K mutation in RSV transcription factor M2‐1, confirming direct binding [248]. Hardened IBs have reduced fluidity, prevented fusion, and restricted component movement—key for viral transcription and CPM/A3E inhibits RSV replication dose‐dependently in mice [248]. For IAV, nucleozins bind NP to form dense, solid aggregates, blocking nuclear accumulation; in vivo, nucleozins improve survival in infected mice. For HIV‐1, efavirenz (EFV)—a first‐generation non‐nucleoside reverse transcriptase inhibitor—induces dense, low‐dynamics reverse transcriptase aggregates, halting reverse transcription [249, 250, 251, 252].

Enhancing host immune condensates strengthens antiviral defenses. PC7A, a polyvalent STING agonist, facilitates the formation of STING condensates and prolongs the activation of innate immunity pathways [255]. Thus, PC7A leads to synergistic therapeutic outcomes in vivo when combined with the STING ligand cGAMP [231]. In MERS‐CoV, the small molecule P3 (5‐benzyloxygramine) binds the NP NTD dimer interface to induce abnormal, nonfunctional NP aggregates that disrupt viral replication. This demonstrates that promoting nonproductive host–viral condensates can also be an effective antiviral strategy [241].

### 6.3 Advanced Modalities: Genetic, Proteolytic, and Biological Approaches

The limited specificity of small molecules has driven the development of advanced, genetically encoded modalities, which aim to precisely manipulate LLPS machinery while minimizing off‐target effects.

Gene therapies, such as CRISPR and small nucleic acid drugs, provide precise tools to modulate proteins that drive or regulate LLPS, such as scaffold proteins, RNA‐binding proteins, molecular chaperones, kinases, and depolymerases. Many viruses exploit these host factors to achieve immune evasion. Cyclophilin A (CypA) is a notable example, as it can be hijacked by HIV, HCV, and SARS. Mechanistic studies have shown that CypA binds PKR, thereby affecting its ability to detect viruses. Therefore, in PKR‐knockout cells generated via CRISPR/Cas9, cyclophilin inhibitors demonstrate only limited efficacy in blocking viral replication. Taken together, these findings suggest that antiviral drugs targeting CypA could provide therapeutic benefit against multiple otherwise difficult‐to‐treat viruses [242, 243].

Nucleic acid drugs, including siRNAs, microRNAs (miRNAs), and antisense oligonucleotides (ASO), effectively reduce the expression of pathogenic proteins by precisely targeting specific mRNA. Miravirsen, an ASO that targets miRNA‐122, has been studied for its potential in treating HCV infection [244, 245]. The stability and immunogenicity of nucleic acid drugs must be optimized through chemical modifications (e.g., a thiophosphate backbone) or novel delivery systems (e.g., GalNAc conjugates) to improve their therapeutic utilization.

Technologies such as CRISPR and ASO allow for the direct knockout or silencing of genes encoding scaffold proteins that drive abnormal phase separation, representing a “grass root” strategy. However, its main challenges are the efficiency of in vivo delivery and the potential for long‐term effects. Emerging protein editing technologies, such as LCB1 peptide N‐glycanases (PNGases), elevate the intervention level from genes and RNA to the protein itself, enabling precise erasure of specific PTMs (such as glycosylation), thereby altering the protein's phase separation ability. This provides an unprecedented tool for directly “rewriting” the host virus interaction interface [246]. Specifically, LCB1, a small peptide that effectively targets the SARS‐CoV‐2 spike protein, was fused with active PNGases to create LCB1–PNGase. Using this protein‐editing tool, N‐linked glycans were removed, and the relevant asparagine sites were converted to aspartate in living mammalian cells. Consequently, syncytia formation was effectively reduced and pseudovirus packaging was inhibited [246, 274]. This technology can be extended to other PTMs (such as ubiquitination and phosphorylation); however, the editing efficiency and off‐target effects remain challenges that require further investigation. Artificial intelligence‐driven protein design platforms, such as AlphaFold, may help accelerate the development of novel, more precise editing tools.

### 6.4 Repurposing Existing Drugs and High‐Throughput Screening

The intricate and dynamic nature of LLPS poses a formidable challenge for conventional small‐molecule drug discovery. To address this, the field is increasingly turning to sophisticated biologics and peptide‐based modalities, which provide greater enhanced specificity for targeting the complex interfaces of biomolecular condensates.

Nanoantibodies offer unique advantages for targeting specific proteins involved in LLPS. Their small size enables deeper tissue penetration and more effective binding to protein targets, which in turn facilitates the modulation of the phase behavior of disease‐associated proteins [275]. TRIM21‐based nanoantibody technology has proven effective in clearing tau protein aggregates in brain cells, restoring cellular function in mice [256]. Furthermore, nanoantibodies with neutralizing activity can inhibit SARS‐CoV‐2 infection by blocking receptor binding at multiple stages of the viral lifecycle [257, 258, 276].

Additionally, in the context of viral infections, interference peptides targeting the dimerization domain of SARS‐NP can disrupt its LLPS, thereby inhibiting viral replication [268]. Wang et al. demonstrated that this domain is essential for SARS2‐NP to undergo LLPS with RNA, which subsequently promotes Lys63‐linked polyubiquitination and aggregation of MAVS to activate the innate antiviral immune response. Peptides designed to disrupt SARS2‐NP LLPS not only inhibited SARS‐CoV‐2 replication but also restored innate antiviral immunity both in vitro and in vivo [268]. These peptides are well tolerated in humans and represent a potent class of antiviral agents targeting LLPS.

In conclusion, the targeted modulation of LLPS presents a promising frontier for antiviral therapy. As our understanding of LLPS in cellular biology expands, it has the potential to transform disease management and enable more effective interventions. However, its clinical translation faces three core challenges: the high overlap between host and viral LLPS mechanisms requires the development of virus‐specific targets (such as NP dimerization domains or SUMO modification sites); the spatiotemporal dynamics of LLPS require precise regulation through reversible small molecules (such as photo controlled drugs) or conditional gene editing; and the delivery bottleneck relies on breakthroughs in nanocarriers (such as liposomes) and organ‐targeting technologies (such as inhaled drug delivery). Future research should focus on multimodal combination therapy (such as kinase inhibitors combined with STING agonists), AI‐driven prediction and design of LLPS interfaces, and rigorous translational validation to advance lead compounds such as GCG and JG40 into clinical trials. Through cross‐disciplinary integration and technological innovation, LLPS‐targeted strategies have the potential to reshape treatment paradigms for numerous infectious diseases.

## 7 Conclusion and Future Perspectives

The study of LLPS has fundamentally transformed our understanding of viral pathogenesis and host immunity. It reveals that the battle between viruses and their hosts is not fought solely with genetic and biochemical mechanisms but also occurs within the physical landscape of biomolecular condensates. This paradigm shift expands our perspective from a purely molecular view to a mesoscale framework, where the assembly, material properties, and functional dynamics of these MLOs are central determinants of infection outcome.

### 7.1 Summarizing the Central Role of LLPS in Virus–Host Conflicts

LLPS serves as a double‐edged sword, co‐opted by both viral invader and host defender. Viruses, as master manipulators of cellular machinery, exploit LLPS to form RFs or IBs. These condensates achieve a remarkable feat: they concentrate viral components like NPs and polymerases to drive efficient genome replication and assembly, while simultaneously excluding or inactivating host antiviral sensors. This spatial organization is not a passive byproduct but an actively regulated strategy for viral immune evasion.

Conversely, the host exploits the same physicochemical principles to mount a robust defense. The formation of innate immune signaling hubs, such as those nucleated by MAVS, RIG‐I, and STING, is driven by LLPS. This enables the rapid, high‐gain amplification of IFN and inflammatory responses. Furthermore, the dynamic regulation of SGs constitutes a critical front in this battle, as viruses have evolved sophisticated strategies to disrupt, repurpose, or inhibit these structures, thereby circumventing translational shutdown and mRNA decay.

Thus, the virus–host interface can be seen as a “battle of condensates”—a contest for control over cellular biophysical space, in which the formation, dissolution, and functional hijacking of phase‐separated assemblies dictate the course of infection.

### 7.2 Key Unresolved Questions and Technological Hurdles

Despite rapid progress, the field remains in its infancy, with several foundational questions still unanswered due to significant technological limitations. A primary challenge is deciphering the precise molecular “coding rules” that govern the functional orientation of condensation bodies, namely, how viral proteins form specific condensates through defined sequences, RNA motifs, and PTMs that selectively recruit viral factors while excluding host components. Another critical hurdle is moving from correlation to causation. While many proteins have been observed in viral or immune condensates, rigorous evidence demonstrating that their phase separation is indispensable for infection is still lacking. Addressing this requires tools capable of perturbing phase separation without disrupting other protein functions. Understanding the spatiotemporal dynamics of condensates in vivo presents an even greater challenge. Current knowledge largely derives from simplified in vitro systems or static snapshots. There is an urgent need to track, in real time, the evolving properties and composition of condensates during live infection, an endeavor that places extreme demands on imaging and biophysical technologies. Ultimately, the core puzzle of host–virus recognition remains unresolved: how do immune sensors accurately distinguish viral from host nucleic acids within the crowded environment of condensates? Solving this question is critical for understanding antiviral specificity and autoimmunity. Addressing these challenges will require technological innovation, including in vivo condensation sensors, real‐time super‐resolution imaging, and chemical biology tools with precise spatiotemporal control, thereby systematically advancing the field toward quantitative and mechanistic precision.

### 7.3 Path Toward LLPS‐Based Antiviral Therapeutics

The therapeutic targeting of LLPS represents a tantalizing yet formidable frontier, whose success hinges on a paradigm shift from targeting single proteins to manipulating the supramolecular architecture of pathogenic condensates. Navigating this path requires a multipronged strategy. A primary challenge is achieving therapeutic specificity, given the shared biophysical principles of host and viral condensates. The solution likely lies in identifying virus‐unique vulnerabilities, such as specific protein–protein interaction interfaces, distinct PTM patterns exploited by the virus, or the unique material properties of viral condensates that could be targeted by small molecules. Given the complexity of the condensate network, embracing multitarget and combination therapies will be crucial. A promising approach involves synergizing a condensate‐disrupting agent, designed to dissolve RFs, with a condensate‐potentiating agent, such as a STING agonist, to enhance immune signaling hubs, thereby simultaneously attacking the virus and bolstering host defenses. The future therapeutic arsenal will also extend beyond traditional small molecules to include advanced modalities. These may encompass engineered nanoantibodies targeting condensate‐specific epitopes, interfering peptides that block critical multivalent interactions, and protein degradation technologies like PROTACs to eliminate scaffold proteins that drive pathogenic LLPS. Successfully navigating this frontier will require a truly cross‐disciplinary effort. Biologists must define the targets, biophysicists must elucidate the material principles, chemists must design the intervention tools, computational scientists must model condensate behavior, and clinicians must guide translational application.

In conclusion, the journey to harness LLPS therapeutically is only beginning. By advancing from descriptive biology to a quantitative and predictive science, we can aspire to develop a new class of “mesoscale medicines” capable of fundamentally disrupting the organizational logic of viral infection, offering hope against even the most elusive and rapidly evolving pathogens.

## Author Contributions

Jiuzhi Xu, Lan Bai, and Bin Wang contributed equally to this work. Jiuzhi Xu and Lan Bai conceived and drafted the manuscript. Jiuzhi Xu and Bin Wang drew the figures. Hai Song, Long Zhang, and Fangfang Zhou provided valuable discussion and revised the manuscript. All authors have read and approved the article.

## Ethics Statement

The authors have nothing to report.

## Conflicts of Interest

The authors declare no conflicts of interest.

## Data Availability

The authors have nothing to report.
